# (Benzoato-κ^2^ *O*,*O*′)(5,5,7,12,12,14-hexa­methyl-1,4,8,11-tetra­azacyclo­tetra­decane-κ^4^ *N*,*N*′,*N*′′,*N*′′′)nickel(II) perchlorate benzoic acid solvate

**DOI:** 10.1107/S1600536808038051

**Published:** 2008-11-22

**Authors:** Guang-Chuan Ou, Min Zhang, Xian-You Yuan, Yong-Qiang Dai

**Affiliations:** a Department of Biology and Chemistry, Hunan University of Science and Engineering, Yongzhou, Hunan 425100, People’s Republic of China

## Abstract

In the title compound, [Ni(C_7_H_5_O_2_)(C_16_H_36_N_4_)]ClO_4_·C_7_H_6_O_2_, the Ni atom displays a distorted octa­hedral coordination geometry with four N atoms of the ligand *rac*-5,5,7,12,12,14-hexa­methyl-1,4,8,11-tetra­azacyclo­tetra­decane (*L*) in a folded configuration and two benzoate (bz) O atoms. The [Ni(*rac-L*)(bz)]^+^ complex cation, perchlorate anion and benzoic acid mol­ecules engage in hydrogen bonding, with N⋯O distances between 2.970 (3) and 3.123 (3) Å and an O⋯O distance of 2.691 (3) Å.

## Related literature

For related background, see: Tait & Busch (1976 ▶); Curtis (1965 ▶). For related structures, see: Ou *et al.* (2008 ▶); Basiuk *et al.* (2001 ▶); Jiang *et al.* (2005 ▶).
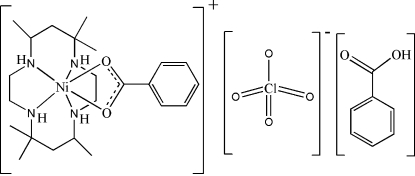


## Experimental

### 

#### Crystal data

[Ni(C_7_H_5_O_2_)(C_16_H_36_N_4_)]ClO_4_·C_7_H_6_O_2_
*M* *_r_* = 685.88
Monoclinic, 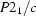

*a* = 8.8035 (11) Å
*b* = 18.138 (2) Å
*c* = 20.966 (3) Å
β = 95.512 (2)°
*V* = 3332.4 (7) Å^3^
*Z* = 4
Mo *K*α radiation
μ = 0.72 mm^−1^
*T* = 293 (2) K
0.48 × 0.26 × 0.15 mm

#### Data collection

Bruker SMART CCD area-detector diffractometer
Absorption correction: multi-scan (*SADABS*; Sheldrick, 1996 ▶) *T*_min_ = 0.725, *T*_max_ = 0.900
22312 measured reflections
7304 independent reflections
5272 reflections with *I* > 2σ(*I*)
*R*_int_ = 0.037

#### Refinement

*R*[*F* ^2^ > 2σ(*F* ^2^)] = 0.042
*wR*(*F* ^2^) = 0.126
*S* = 1.11
7304 reflections
404 parameters
H-atom parameters constrained
Δρ_max_ = 0.58 e Å^−3^
Δρ_min_ = −0.42 e Å^−3^

### 

Data collection: *SMART* (Bruker, 1997 ▶); cell refinement: *SAINT* (Bruker, 2003 ▶); data reduction: *SAINT*; program(s) used to solve structure: *SHELXS97* (Sheldrick, 2008 ▶); program(s) used to refine structure: *SHELXL97* (Sheldrick, 2008 ▶); molecular graphics: *SHELXTL* (Sheldrick, 2008 ▶); software used to prepare material for publication: *SHELXTL*.

## Supplementary Material

Crystal structure: contains datablocks I, global. DOI: 10.1107/S1600536808038051/pv2117sup1.cif

Structure factors: contains datablocks I. DOI: 10.1107/S1600536808038051/pv2117Isup2.hkl

Additional supplementary materials:  crystallographic information; 3D view; checkCIF report

## Figures and Tables

**Table 1:** Hydrogen-bond geometry (Å, °)

*D*—H⋯*A*	*D*—H	H⋯*A*	*D*⋯*A*	*D*—H⋯*A*
N1—H1*C*⋯O4^i^	0.91	2.07	2.970 (3)	171
N4—H4*D*⋯O6^ii^	0.91	2.13	3.001 (3)	161
O3—H3*B*⋯O1^iii^	0.82	1.87	2.691 (3)	174
N3—H3*C*⋯O8	0.91	2.22	3.108 (3)	166
N2—H2*C*⋯O6^ii^	0.91	2.25	3.123 (3)	160

Symmetry codes: (i) 
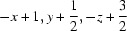
; (ii) 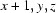; (iii) 
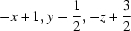
.

**Fig. 1.**
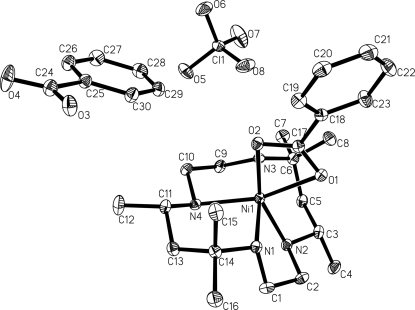
The molecular structure of (I), showing displacement ellipsoids at the 30% probability level; H-atoms have been excluded for clarity.

**Fig. 2.**
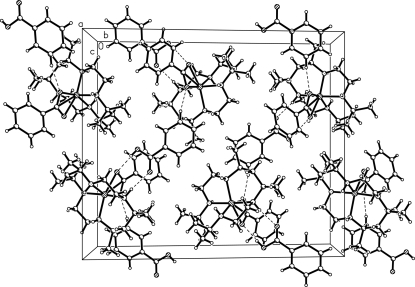
A view of the packing of the title compound along a axis.

## supplementary crystallographic information

### Comment

It is important to control the geometries of *ML*^2+^ [*M* = Ni(II), Co(II), Cu(II)] with *cis*- or *trans*-conformation, since they form different structures and show different properties (Tait & Busch, 1976; Curtis, 1965). Continuing our research (Ou *et al.*, 2008), we have synthesized the title compound, (I), which is presented in this paper.

The asymmetric unit of the title compouind, (I), contains one [Ni(*rac*-*L*)(bz)]^+^ cation, one [ClO_4_]^-^ anion and one benzoic acid molecule (Fig. 1). The six-coordinated Ni^2+^ of the complex [Ni(*rac*-*L*)(bz)]^+^ cation displays a distorted octahedral geometry by coordination with four nitrogen atoms of the macrocyclic ligand *L* in a folded configuration, and two carboxylate oxygen atoms of benzoic acid in *cis*-position. The Ni—N distances range between 2.082 (2) to 2.134 (2) Å, and are slightly shorter than the Ni—O distance (2.116 (2) and 2.212 (2) Å). The neighbouring cations, anions and benzoic acid are connected to each other through intermolecular hydrogen bond of the types N—H···O and O—H···O (Table 1, Fig. 2). The crystal structures of a few compound closely related to (I) have been reported (Ou *et al.*, 2008*a*,*b*; Basiuk *et al.* 2001; Jiang *et al.*,  2005).

### Experimental

Benzoic acid (0.36 g, 3 mmol) and NaOH (0.08 g, 2 mmol) were dissolved in 15 ml of water. To this solution was added [Ni(rac-*L*)](ClO_4_)_2_ (0.54 g, 1 mmol) dissolved in 2 ml of CH_3_CN. The solution was left to stand at room temperature and blue crystals formed after several weeks.

### Refinement

H atoms bound to C, O and N atoms were positioned geometrically and refined using the riding model, and with C—H = 0.93, 0.96, 0.97 and 0.98 Å, for aryl, methyl, methylene and methine H-atoms, O—H = 0.82 Å and N—H = 0.91 Å, and with *U_iso_*(H) set to 1.5*U*_eq_(methyl C) and 1.2*U*_eq_(the rest of the parent atoms).

### Crystal data

**Table:**

[Ni(C_7_H_5_O_2_)(C_16_H_36_N_4_)]ClO_4_·C_7_H_6_O_2_	*F*_000_ = 1456
*M**_r_* = 685.88	*D*_x_ = 1.367 Mg m^−^^3^
Monoclinic, *P*2_1_/*c*	Mo *K*α radiation λ = 0.71073 Å
*a* = 8.8035 (11) Å	Cell parameters from 7647 reflections
*b* = 18.138 (2) Å	θ = 2.3–26.9º
*c* = 20.966 (3) Å	µ = 0.72 mm^−^^1^
β = 95.512 (2)º	*T* = 293 (2) K
*V* = 3332.4 (7) Å^3^	Prism, light-blue
*Z* = 4	0.48 × 0.26 × 0.15 mm

### Data collection

**Table:**

Bruker SMART CCD area-detector diffractometer	7304 independent reflections
Radiation source: fine-focus sealed tube	5272 reflections with *I* > 2σ(*I*)
Monochromator: graphite	*R*_int_ = 0.037
*T* = 293(2) K	θ_max_ = 27.1º
φ and ω scans	θ_min_ = 1.5º
Absorption correction: multi-scan(SADABS; Sheldrick, 1996)	*h* = −10→11
*T*_min_ = 0.725, *T*_max_ = 0.900	*k* = −20→23
22312 measured reflections	*l* = −26→26

### Refinement

**Table:**

Refinement on *F*^2^	Secondary atom site location: difference Fourier map
Least-squares matrix: full	Hydrogen site location: inferred from neighbouring sites
*R*[*F*^2^ > 2σ(*F*^2^)] = 0.042	H-atom parameters constrained
*wR*(*F*^2^) = 0.126	*w* = 1/[σ^2^(*F*_o_^2^) + (0.0643*P*)^2^ + 0.7856*P*] where *P* = (*F*_o_^2^ + 2*F*_c_^2^)/3
*S* = 1.11	(Δ/σ)_max_ = 0.001
7304 reflections	Δρ_max_ = 0.58 e Å^−^^3^
404 parameters	Δρ_min_ = −0.42 e Å^−^^3^
Primary atom site location: structure-invariant direct methods	Extinction correction: none

### Special details

**Table:**

Geometry. All e.s.d.'s (except the e.s.d. in the dihedral angle between two l.s. planes) are estimated using the full covariance matrix. The cell e.s.d.'s are taken into account individually in the estimation of e.s.d.'s in distances, angles and torsion angles; correlations between e.s.d.'s in cell parameters are only used when they are defined by crystal symmetry. An approximate (isotropic) treatment of cell e.s.d.'s is used for estimating e.s.d.'s involving l.s. planes.
Refinement. Refinement of *F*^2^ against ALL reflections. The weighted *R*-factor *wR* and goodness of fit *S* are based on *F*^2^, conventional *R*-factors *R* are based on *F*, with *F* set to zero for negative *F*^2^. The threshold expression of *F*^2^ > σ(*F*^2^) is used only for calculating *R*-factors(gt) *etc*. and is not relevant to the choice of reflections for refinement. *R*-factors based on *F*^2^ are statistically about twice as large as those based on *F*, and *R*- factors based on ALL data will be even larger.

### Fractional atomic coordinates and isotropic or equivalent isotropic displacement parameters (Å^2^)

**Table:**

	*x*	*y*	*z*	*U*_iso_*/*U*_eq_	
Ni1	0.64844 (4)	0.771339 (17)	0.564296 (14)	0.01920 (11)	
Cl1	0.08075 (7)	0.70176 (4)	0.44238 (3)	0.02611 (16)	
N3	0.5075 (2)	0.77519 (11)	0.47565 (9)	0.0208 (5)	
H3C	0.4098	0.7716	0.4861	0.025*	
N1	0.8063 (2)	0.76085 (12)	0.64759 (9)	0.0219 (5)	
H1C	0.7838	0.7983	0.6740	0.026*	
O1	0.5623 (2)	0.87209 (10)	0.60812 (8)	0.0226 (4)	
O2	0.4528 (2)	0.76422 (10)	0.61520 (8)	0.0253 (4)	
N4	0.6736 (2)	0.65959 (12)	0.54328 (10)	0.0220 (5)	
H4D	0.7638	0.6540	0.5260	0.026*	
O6	−0.0503 (3)	0.67518 (13)	0.47084 (12)	0.0527 (6)	
O5	0.1734 (3)	0.64124 (12)	0.42671 (10)	0.0428 (6)	
N2	0.8262 (2)	0.82271 (12)	0.52275 (10)	0.0233 (5)	
H2C	0.8728	0.7876	0.5005	0.028*	
O8	0.1626 (3)	0.74917 (15)	0.48804 (11)	0.0530 (6)	
C6	0.5101 (3)	0.84150 (15)	0.43238 (12)	0.0258 (6)	
C14	0.8130 (3)	0.69224 (14)	0.68843 (12)	0.0244 (6)	
C9	0.5394 (3)	0.70458 (14)	0.44370 (12)	0.0257 (6)	
H9A	0.6351	0.7081	0.4245	0.031*	
H9B	0.4589	0.6943	0.4100	0.031*	
C18	0.3568 (3)	0.86310 (14)	0.67420 (11)	0.0229 (6)	
C17	0.4615 (3)	0.83124 (14)	0.62995 (11)	0.0217 (5)	
O7	0.0320 (4)	0.74181 (15)	0.38604 (11)	0.0672 (8)	
C7	0.4224 (3)	0.82719 (17)	0.36663 (12)	0.0326 (7)	
H7A	0.4733	0.7894	0.3447	0.049*	
H7B	0.4187	0.8717	0.3418	0.049*	
H7C	0.3204	0.8115	0.3724	0.049*	
C3	0.7870 (3)	0.88481 (15)	0.47783 (12)	0.0278 (6)	
H3A	0.7378	0.9235	0.5011	0.033*	
C5	0.6758 (3)	0.86021 (15)	0.42156 (12)	0.0268 (6)	
H5A	0.7193	0.8170	0.4030	0.032*	
H5B	0.6727	0.8989	0.3895	0.032*	
C23	0.3032 (4)	0.93478 (16)	0.66577 (14)	0.0359 (7)	
H23	0.3329	0.9634	0.6323	0.043*	
C15	0.6751 (3)	0.69292 (16)	0.72747 (13)	0.0317 (7)	
H15A	0.6786	0.7362	0.7540	0.048*	
H15B	0.6770	0.6497	0.7540	0.048*	
H15C	0.5830	0.6933	0.6989	0.048*	
C10	0.5483 (3)	0.64341 (15)	0.49227 (12)	0.0265 (6)	
H10A	0.4521	0.6393	0.5110	0.032*	
H10B	0.5679	0.5969	0.4717	0.032*	
C1	0.9551 (3)	0.78073 (16)	0.62444 (13)	0.0289 (6)	
H1A	0.9963	0.7385	0.6035	0.035*	
H1B	1.0269	0.7944	0.6605	0.035*	
C2	0.9351 (3)	0.84413 (16)	0.57805 (12)	0.0286 (6)	
H2A	0.8965	0.8868	0.5992	0.034*	
H2B	1.0327	0.8571	0.5632	0.034*	
C13	0.8127 (3)	0.62333 (15)	0.64645 (12)	0.0280 (6)	
H13A	0.9016	0.6257	0.6225	0.034*	
H13B	0.8261	0.5809	0.6746	0.034*	
C8	0.4328 (4)	0.90494 (16)	0.46373 (13)	0.0334 (7)	
H8A	0.3276	0.8929	0.4669	0.050*	
H8B	0.4390	0.9486	0.4383	0.050*	
H8C	0.4830	0.9134	0.5058	0.050*	
C19	0.3146 (3)	0.82170 (16)	0.72532 (12)	0.0298 (6)	
H19	0.3495	0.7735	0.7311	0.036*	
C4	0.9287 (4)	0.91798 (16)	0.45124 (14)	0.0373 (7)	
H4A	1.0007	0.9329	0.4861	0.056*	
H4B	0.8992	0.9600	0.4251	0.056*	
H4C	0.9747	0.8817	0.4258	0.056*	
C16	0.9575 (3)	0.69124 (16)	0.73619 (13)	0.0346 (7)	
H16A	1.0463	0.6881	0.7131	0.052*	
H16B	0.9543	0.6493	0.7640	0.052*	
H16C	0.9619	0.7356	0.7613	0.052*	
C11	0.6742 (3)	0.60875 (14)	0.59886 (12)	0.0260 (6)	
H11	0.5826	0.6189	0.6205	0.031*	
C12	0.6712 (4)	0.52789 (16)	0.58023 (15)	0.0436 (8)	
H12A	0.5821	0.5181	0.5513	0.065*	
H12B	0.6685	0.4981	0.6179	0.065*	
H12C	0.7610	0.5163	0.5596	0.065*	
C20	0.2205 (3)	0.85191 (18)	0.76787 (13)	0.0365 (7)	
H20	0.1938	0.8244	0.8025	0.044*	
C22	0.2050 (4)	0.96368 (19)	0.70754 (15)	0.0443 (8)	
H22	0.1651	1.0108	0.7008	0.053*	
C21	0.1668 (4)	0.92287 (19)	0.75872 (15)	0.0423 (8)	
H21	0.1043	0.9432	0.7874	0.051*	
O3	0.3369 (3)	0.47611 (10)	0.80658 (9)	0.0360 (5)	
H3B	0.3730	0.4465	0.8334	0.054*	
O4	0.2469 (3)	0.37507 (12)	0.75552 (10)	0.0530 (7)	
C30	0.2927 (3)	0.55960 (15)	0.69518 (13)	0.0303 (6)	
H30	0.3393	0.5819	0.7319	0.036*	
C29	0.2669 (3)	0.59937 (16)	0.63842 (14)	0.0342 (7)	
H29	0.2966	0.6485	0.6370	0.041*	
C24	0.2768 (3)	0.44005 (16)	0.75521 (13)	0.0308 (6)	
C25	0.2481 (3)	0.48619 (15)	0.69640 (12)	0.0264 (6)	
C26	0.1793 (3)	0.45290 (16)	0.64188 (13)	0.0320 (7)	
H26	0.1509	0.4036	0.6429	0.038*	
C27	0.1524 (4)	0.49276 (18)	0.58565 (13)	0.0362 (7)	
H27	0.1046	0.4706	0.5491	0.043*	
C28	0.1971 (4)	0.56580 (17)	0.58414 (14)	0.0365 (7)	
H28	0.1801	0.5925	0.5463	0.044*	

### Atomic displacement parameters (Å^2^)

**Table:**

	*U*^11^	*U*^22^	*U*^33^	*U*^12^	*U*^13^	*U*^23^
Ni1	0.02147 (19)	0.01929 (18)	0.01704 (16)	−0.00093 (13)	0.00287 (12)	−0.00228 (13)
Cl1	0.0256 (4)	0.0316 (4)	0.0216 (3)	0.0031 (3)	0.0049 (3)	0.0003 (3)
N3	0.0188 (11)	0.0249 (12)	0.0193 (10)	−0.0003 (9)	0.0048 (8)	0.0003 (9)
N1	0.0241 (12)	0.0218 (12)	0.0198 (10)	0.0002 (9)	0.0028 (9)	−0.0028 (9)
O1	0.0251 (10)	0.0244 (10)	0.0187 (8)	−0.0022 (8)	0.0044 (7)	−0.0002 (7)
O2	0.0289 (11)	0.0240 (10)	0.0233 (9)	−0.0022 (8)	0.0044 (8)	−0.0030 (8)
N4	0.0210 (12)	0.0240 (12)	0.0214 (10)	−0.0026 (9)	0.0035 (9)	−0.0034 (9)
O6	0.0437 (14)	0.0526 (15)	0.0674 (16)	−0.0160 (11)	0.0336 (12)	−0.0186 (12)
O5	0.0486 (14)	0.0445 (13)	0.0372 (11)	0.0199 (11)	0.0146 (10)	0.0008 (10)
N2	0.0251 (12)	0.0221 (12)	0.0231 (11)	−0.0010 (9)	0.0038 (9)	−0.0029 (9)
O8	0.0355 (14)	0.0714 (17)	0.0527 (14)	−0.0149 (12)	0.0072 (11)	−0.0293 (12)
C6	0.0291 (15)	0.0268 (15)	0.0212 (12)	−0.0008 (11)	0.0008 (11)	0.0031 (11)
C14	0.0281 (15)	0.0251 (14)	0.0199 (12)	0.0029 (11)	0.0015 (11)	0.0002 (11)
C9	0.0325 (16)	0.0257 (14)	0.0185 (12)	−0.0047 (12)	−0.0003 (11)	−0.0047 (10)
C18	0.0221 (14)	0.0271 (14)	0.0195 (12)	−0.0008 (11)	0.0013 (10)	−0.0017 (10)
C17	0.0244 (14)	0.0240 (14)	0.0157 (11)	0.0014 (11)	−0.0024 (10)	0.0012 (10)
O7	0.107 (2)	0.0608 (17)	0.0328 (12)	0.0366 (16)	−0.0002 (14)	0.0145 (12)
C7	0.0348 (17)	0.0392 (17)	0.0230 (13)	−0.0028 (13)	−0.0018 (12)	0.0060 (12)
C3	0.0344 (16)	0.0204 (14)	0.0288 (14)	−0.0024 (12)	0.0044 (12)	−0.0001 (11)
C5	0.0337 (16)	0.0236 (14)	0.0234 (13)	−0.0027 (12)	0.0049 (11)	0.0051 (11)
C23	0.0443 (19)	0.0305 (17)	0.0345 (15)	0.0063 (14)	0.0118 (14)	−0.0006 (13)
C15	0.0438 (18)	0.0303 (16)	0.0222 (13)	0.0037 (13)	0.0090 (12)	0.0011 (12)
C10	0.0269 (15)	0.0271 (15)	0.0247 (13)	−0.0062 (11)	−0.0011 (11)	−0.0037 (11)
C1	0.0210 (14)	0.0395 (17)	0.0258 (13)	−0.0041 (12)	0.0006 (11)	0.0002 (12)
C2	0.0248 (15)	0.0353 (16)	0.0251 (13)	−0.0086 (12)	0.0001 (11)	−0.0050 (12)
C13	0.0324 (16)	0.0252 (15)	0.0261 (13)	0.0028 (12)	0.0016 (11)	−0.0012 (11)
C8	0.0375 (18)	0.0328 (16)	0.0293 (14)	0.0075 (13)	0.0000 (12)	0.0042 (12)
C19	0.0336 (17)	0.0337 (16)	0.0222 (13)	−0.0015 (13)	0.0037 (12)	−0.0006 (12)
C4	0.0380 (18)	0.0305 (17)	0.0433 (17)	−0.0106 (13)	0.0030 (14)	0.0065 (14)
C16	0.0395 (18)	0.0364 (17)	0.0259 (14)	0.0026 (14)	−0.0071 (12)	−0.0011 (12)
C11	0.0326 (16)	0.0211 (14)	0.0246 (13)	−0.0028 (11)	0.0040 (11)	0.0009 (11)
C12	0.069 (2)	0.0228 (16)	0.0367 (16)	−0.0051 (15)	−0.0085 (16)	0.0017 (13)
C20	0.0347 (17)	0.050 (2)	0.0263 (14)	−0.0108 (14)	0.0100 (12)	−0.0080 (13)
C22	0.045 (2)	0.0380 (19)	0.0514 (19)	0.0114 (15)	0.0147 (16)	−0.0105 (15)
C21	0.0358 (18)	0.056 (2)	0.0379 (17)	−0.0041 (16)	0.0158 (14)	−0.0217 (15)
O3	0.0535 (14)	0.0279 (11)	0.0243 (10)	0.0026 (10)	−0.0078 (9)	0.0011 (8)
O4	0.093 (2)	0.0331 (13)	0.0289 (11)	−0.0227 (12)	−0.0128 (12)	0.0130 (9)
C30	0.0327 (16)	0.0280 (15)	0.0297 (14)	−0.0047 (12)	0.0005 (12)	0.0028 (12)
C29	0.0327 (17)	0.0253 (15)	0.0455 (17)	0.0006 (12)	0.0085 (13)	0.0099 (13)
C24	0.0365 (17)	0.0299 (16)	0.0255 (14)	−0.0018 (13)	0.0010 (12)	0.0037 (12)
C25	0.0286 (16)	0.0257 (15)	0.0250 (13)	−0.0016 (11)	0.0028 (11)	0.0038 (11)
C26	0.0370 (18)	0.0307 (16)	0.0283 (14)	−0.0033 (13)	0.0032 (12)	0.0034 (12)
C27	0.0414 (19)	0.0423 (18)	0.0244 (13)	−0.0005 (14)	0.0005 (12)	0.0038 (13)
C28	0.0374 (18)	0.0431 (19)	0.0297 (15)	0.0064 (14)	0.0068 (13)	0.0152 (13)

### Geometric parameters (Å, °)

**Table:**

Ni1—N2	2.082 (2)	C15—H15C	0.9600
Ni1—N4	2.091 (2)	C10—H10A	0.9700
Ni1—O2	2.116 (2)	C10—H10B	0.9700
Ni1—N1	2.133 (2)	C1—C2	1.506 (4)
Ni1—N3	2.134 (2)	C1—H1A	0.9700
Ni1—O1	2.212 (2)	C1—H1B	0.9700
Cl1—O7	1.418 (2)	C2—H2A	0.9700
Cl1—O5	1.425 (2)	C2—H2B	0.9700
Cl1—O8	1.429 (2)	C13—C11	1.523 (4)
Cl1—O6	1.433 (2)	C13—H13A	0.9700
N3—C9	1.484 (3)	C13—H13B	0.9700
N3—C6	1.508 (3)	C8—H8A	0.9600
N3—H3C	0.9100	C8—H8B	0.9600
N1—C1	1.485 (3)	C8—H8C	0.9600
N1—C14	1.508 (3)	C19—C20	1.387 (4)
N1—H1C	0.9100	C19—H19	0.9300
O1—C17	1.274 (3)	C4—H4A	0.9600
O2—C17	1.255 (3)	C4—H4B	0.9600
N4—C11	1.485 (3)	C4—H4C	0.9600
N4—C10	1.490 (3)	C16—H16A	0.9600
N4—H4D	0.9100	C16—H16B	0.9600
N2—C2	1.484 (3)	C16—H16C	0.9600
N2—C3	1.487 (3)	C11—C12	1.517 (4)
N2—H2C	0.9100	C11—H11	0.9800
C6—C8	1.518 (4)	C12—H12A	0.9600
C6—C5	1.536 (4)	C12—H12B	0.9600
C6—C7	1.536 (3)	C12—H12C	0.9600
C14—C13	1.529 (4)	C20—C21	1.378 (5)
C14—C15	1.529 (4)	C20—H20	0.9300
C14—C16	1.541 (4)	C22—C21	1.372 (5)
C9—C10	1.503 (4)	C22—H22	0.9300
C9—H9A	0.9700	C21—H21	0.9300
C9—H9B	0.9700	O3—C24	1.326 (3)
C18—C19	1.388 (4)	O3—H3B	0.8200
C18—C23	1.389 (4)	O4—C24	1.208 (3)
C18—C17	1.486 (4)	C30—C25	1.389 (4)
C7—H7A	0.9600	C30—C29	1.392 (4)
C7—H7B	0.9600	C30—H30	0.9300
C7—H7C	0.9600	C29—C28	1.382 (4)
C3—C5	1.526 (4)	C29—H29	0.9300
C3—C4	1.537 (4)	C24—C25	1.492 (4)
C3—H3A	0.9800	C25—C26	1.380 (4)
C5—H5A	0.9700	C26—C27	1.384 (4)
C5—H5B	0.9700	C26—H26	0.9300
C23—C22	1.391 (4)	C27—C28	1.383 (4)
C23—H23	0.9300	C27—H27	0.9300
C15—H15A	0.9600	C28—H28	0.9300
C15—H15B	0.9600		
			
N2—Ni1—N4	104.28 (8)	C14—C15—H15B	109.5
N2—Ni1—O2	156.80 (8)	H15A—C15—H15B	109.5
N4—Ni1—O2	98.91 (8)	C14—C15—H15C	109.5
N2—Ni1—N1	85.74 (8)	H15A—C15—H15C	109.5
N4—Ni1—N1	90.70 (8)	H15B—C15—H15C	109.5
O2—Ni1—N1	94.56 (8)	N4—C10—C9	109.1 (2)
N2—Ni1—N3	91.05 (8)	N4—C10—H10A	109.9
N4—Ni1—N3	84.99 (8)	C9—C10—H10A	109.9
O2—Ni1—N3	90.46 (8)	N4—C10—H10B	109.9
N1—Ni1—N3	173.84 (8)	C9—C10—H10B	109.9
N2—Ni1—O1	96.06 (8)	H10A—C10—H10B	108.3
N4—Ni1—O1	159.39 (8)	N1—C1—C2	110.1 (2)
O2—Ni1—O1	60.82 (7)	N1—C1—H1A	109.6
N1—Ni1—O1	87.49 (7)	C2—C1—H1A	109.6
N3—Ni1—O1	98.09 (7)	N1—C1—H1B	109.6
O7—Cl1—O5	109.69 (14)	C2—C1—H1B	109.6
O7—Cl1—O8	110.04 (18)	H1A—C1—H1B	108.1
O5—Cl1—O8	110.78 (15)	N2—C2—C1	109.4 (2)
O7—Cl1—O6	109.14 (18)	N2—C2—H2A	109.8
O5—Cl1—O6	109.81 (14)	C1—C2—H2A	109.8
O8—Cl1—O6	107.34 (14)	N2—C2—H2B	109.8
C9—N3—C6	113.71 (19)	C1—C2—H2B	109.8
C9—N3—Ni1	104.21 (14)	H2A—C2—H2B	108.2
C6—N3—Ni1	120.59 (15)	C11—C13—C14	118.4 (2)
C9—N3—H3C	105.7	C11—C13—H13A	107.7
C6—N3—H3C	105.7	C14—C13—H13A	107.7
Ni1—N3—H3C	105.7	C11—C13—H13B	107.7
C1—N1—C14	113.5 (2)	C14—C13—H13B	107.7
C1—N1—Ni1	103.69 (15)	H13A—C13—H13B	107.1
C14—N1—Ni1	121.68 (15)	C6—C8—H8A	109.5
C1—N1—H1C	105.6	C6—C8—H8B	109.5
C14—N1—H1C	105.6	H8A—C8—H8B	109.5
Ni1—N1—H1C	105.6	C6—C8—H8C	109.5
C17—O1—Ni1	86.98 (15)	H8A—C8—H8C	109.5
C17—O2—Ni1	91.82 (16)	H8B—C8—H8C	109.5
C11—N4—C10	112.8 (2)	C20—C19—C18	120.2 (3)
C11—N4—Ni1	115.40 (15)	C20—C19—H19	119.9
C10—N4—Ni1	104.85 (15)	C18—C19—H19	119.9
C11—N4—H4D	107.8	C3—C4—H4A	109.5
C10—N4—H4D	107.8	C3—C4—H4B	109.5
Ni1—N4—H4D	107.8	H4A—C4—H4B	109.5
C2—N2—C3	112.9 (2)	C3—C4—H4C	109.5
C2—N2—Ni1	104.20 (15)	H4A—C4—H4C	109.5
C3—N2—Ni1	117.77 (16)	H4B—C4—H4C	109.5
C2—N2—H2C	107.1	C14—C16—H16A	109.5
C3—N2—H2C	107.1	C14—C16—H16B	109.5
Ni1—N2—H2C	107.1	H16A—C16—H16B	109.5
N3—C6—C8	108.1 (2)	C14—C16—H16C	109.5
N3—C6—C5	109.5 (2)	H16A—C16—H16C	109.5
C8—C6—C5	111.6 (2)	H16B—C16—H16C	109.5
N3—C6—C7	111.7 (2)	N4—C11—C12	113.5 (2)
C8—C6—C7	108.0 (2)	N4—C11—C13	110.4 (2)
C5—C6—C7	107.9 (2)	C12—C11—C13	109.2 (2)
N1—C14—C13	110.5 (2)	N4—C11—H11	107.8
N1—C14—C15	107.9 (2)	C12—C11—H11	107.8
C13—C14—C15	110.9 (2)	C13—C11—H11	107.8
N1—C14—C16	111.3 (2)	C11—C12—H12A	109.5
C13—C14—C16	108.7 (2)	C11—C12—H12B	109.5
C15—C14—C16	107.5 (2)	H12A—C12—H12B	109.5
N3—C9—C10	109.3 (2)	C11—C12—H12C	109.5
N3—C9—H9A	109.8	H12A—C12—H12C	109.5
C10—C9—H9A	109.8	H12B—C12—H12C	109.5
N3—C9—H9B	109.8	C21—C20—C19	119.8 (3)
C10—C9—H9B	109.8	C21—C20—H20	120.1
H9A—C9—H9B	108.3	C19—C20—H20	120.1
C19—C18—C23	119.5 (3)	C21—C22—C23	120.2 (3)
C19—C18—C17	120.0 (2)	C21—C22—H22	119.9
C23—C18—C17	120.5 (2)	C23—C22—H22	119.9
O2—C17—O1	120.2 (2)	C22—C21—C20	120.4 (3)
O2—C17—C18	120.2 (2)	C22—C21—H21	119.8
O1—C17—C18	119.6 (2)	C20—C21—H21	119.8
C6—C7—H7A	109.5	C24—O3—H3B	109.5
C6—C7—H7B	109.5	C25—C30—C29	119.3 (3)
H7A—C7—H7B	109.5	C25—C30—H30	120.3
C6—C7—H7C	109.5	C29—C30—H30	120.3
H7A—C7—H7C	109.5	C28—C29—C30	119.9 (3)
H7B—C7—H7C	109.5	C28—C29—H29	120.0
N2—C3—C5	111.1 (2)	C30—C29—H29	120.0
N2—C3—C4	112.3 (2)	O4—C24—O3	123.2 (2)
C5—C3—C4	108.5 (2)	O4—C24—C25	122.2 (2)
N2—C3—H3A	108.3	O3—C24—C25	114.7 (2)
C5—C3—H3A	108.3	C26—C25—C30	120.4 (2)
C4—C3—H3A	108.3	C26—C25—C24	117.7 (2)
C3—C5—C6	119.9 (2)	C30—C25—C24	121.8 (2)
C3—C5—H5A	107.3	C25—C26—C27	120.2 (3)
C6—C5—H5A	107.3	C25—C26—H26	119.9
C3—C5—H5B	107.3	C27—C26—H26	119.9
C6—C5—H5B	107.3	C28—C27—C26	119.6 (3)
H5A—C5—H5B	106.9	C28—C27—H27	120.2
C18—C23—C22	119.8 (3)	C26—C27—H27	120.2
C18—C23—H23	120.1	C29—C28—C27	120.5 (3)
C22—C23—H23	120.1	C29—C28—H28	119.7
C14—C15—H15A	109.5	C27—C28—H28	119.7
			
N2—Ni1—N3—C9	90.62 (16)	Ni1—N1—C14—C15	−74.8 (2)
N4—Ni1—N3—C9	−13.62 (16)	C1—N1—C14—C16	42.7 (3)
O2—Ni1—N3—C9	−112.53 (16)	Ni1—N1—C14—C16	167.49 (17)
N1—Ni1—N3—C9	32.1 (8)	C6—N3—C9—C10	174.9 (2)
O1—Ni1—N3—C9	−173.10 (15)	Ni1—N3—C9—C10	41.7 (2)
N2—Ni1—N3—C6	−38.55 (19)	Ni1—O2—C17—O1	4.4 (2)
N4—Ni1—N3—C6	−142.79 (19)	Ni1—O2—C17—C18	−174.22 (19)
O2—Ni1—N3—C6	118.30 (18)	Ni1—O1—C17—O2	−4.2 (2)
N1—Ni1—N3—C6	−97.1 (8)	Ni1—O1—C17—C18	174.4 (2)
O1—Ni1—N3—C6	57.73 (19)	C19—C18—C17—O2	36.7 (4)
N2—Ni1—N1—C1	−11.37 (16)	C23—C18—C17—O2	−144.8 (3)
N4—Ni1—N1—C1	92.90 (16)	C19—C18—C17—O1	−141.9 (2)
O2—Ni1—N1—C1	−168.10 (16)	C23—C18—C17—O1	36.6 (4)
N3—Ni1—N1—C1	47.4 (8)	C2—N2—C3—C5	179.6 (2)
O1—Ni1—N1—C1	−107.64 (16)	Ni1—N2—C3—C5	−58.8 (3)
N2—Ni1—N1—C14	−140.56 (19)	C2—N2—C3—C4	57.9 (3)
N4—Ni1—N1—C14	−36.29 (19)	Ni1—N2—C3—C4	179.49 (17)
O2—Ni1—N1—C14	62.71 (19)	N2—C3—C5—C6	70.1 (3)
N3—Ni1—N1—C14	−81.8 (8)	C4—C3—C5—C6	−166.0 (2)
O1—Ni1—N1—C14	123.17 (18)	N3—C6—C5—C3	−64.4 (3)
N2—Ni1—O1—C17	−179.64 (13)	C8—C6—C5—C3	55.3 (3)
N4—Ni1—O1—C17	−8.9 (3)	C7—C6—C5—C3	173.8 (2)
O2—Ni1—O1—C17	2.45 (13)	C19—C18—C23—C22	−1.6 (4)
N1—Ni1—O1—C17	−94.19 (14)	C17—C18—C23—C22	179.9 (3)
N3—Ni1—O1—C17	88.43 (14)	C11—N4—C10—C9	170.4 (2)
N2—Ni1—O2—C17	−7.8 (3)	Ni1—N4—C10—C9	44.0 (2)
N4—Ni1—O2—C17	173.50 (14)	N3—C9—C10—N4	−60.2 (3)
N1—Ni1—O2—C17	82.07 (15)	C14—N1—C1—C2	173.4 (2)
N3—Ni1—O2—C17	−101.50 (14)	Ni1—N1—C1—C2	39.4 (2)
O1—Ni1—O2—C17	−2.48 (13)	C3—N2—C2—C1	173.9 (2)
N2—Ni1—N4—C11	129.24 (18)	Ni1—N2—C2—C1	45.0 (2)
O2—Ni1—N4—C11	−51.27 (18)	N1—C1—C2—N2	−59.7 (3)
N1—Ni1—N4—C11	43.46 (18)	N1—C14—C13—C11	−62.5 (3)
N3—Ni1—N4—C11	−140.95 (18)	C15—C14—C13—C11	57.1 (3)
O1—Ni1—N4—C11	−41.3 (3)	C16—C14—C13—C11	175.1 (2)
N2—Ni1—N4—C10	−106.03 (16)	C23—C18—C19—C20	−0.4 (4)
O2—Ni1—N4—C10	73.46 (16)	C17—C18—C19—C20	178.1 (2)
N1—Ni1—N4—C10	168.18 (16)	C10—N4—C11—C12	51.8 (3)
N3—Ni1—N4—C10	−16.22 (16)	Ni1—N4—C11—C12	172.2 (2)
O1—Ni1—N4—C10	83.5 (3)	C10—N4—C11—C13	174.8 (2)
N4—Ni1—N2—C2	−107.64 (16)	Ni1—N4—C11—C13	−64.7 (2)
O2—Ni1—N2—C2	73.6 (3)	C14—C13—C11—N4	74.4 (3)
N1—Ni1—N2—C2	−18.00 (16)	C14—C13—C11—C12	−160.1 (2)
N3—Ni1—N2—C2	167.26 (16)	C18—C19—C20—C21	1.1 (4)
O1—Ni1—N2—C2	69.01 (16)	C18—C23—C22—C21	3.1 (5)
N4—Ni1—N2—C3	126.38 (17)	C23—C22—C21—C20	−2.5 (5)
O2—Ni1—N2—C3	−52.3 (3)	C19—C20—C21—C22	0.4 (5)
N1—Ni1—N2—C3	−143.98 (18)	C25—C30—C29—C28	0.2 (4)
N3—Ni1—N2—C3	41.29 (18)	C29—C30—C25—C26	0.2 (4)
O1—Ni1—N2—C3	−56.96 (18)	C29—C30—C25—C24	178.1 (3)
C9—N3—C6—C8	163.9 (2)	O4—C24—C25—C26	2.7 (5)
Ni1—N3—C6—C8	−71.2 (2)	O3—C24—C25—C26	−177.2 (3)
C9—N3—C6—C5	−74.3 (3)	O4—C24—C25—C30	−175.3 (3)
Ni1—N3—C6—C5	50.6 (3)	O3—C24—C25—C30	4.8 (4)
C9—N3—C6—C7	45.2 (3)	C30—C25—C26—C27	−0.8 (4)
Ni1—N3—C6—C7	170.07 (17)	C24—C25—C26—C27	−178.8 (3)
C1—N1—C14—C13	−78.2 (3)	C25—C26—C27—C28	1.0 (5)
Ni1—N1—C14—C13	46.7 (3)	C30—C29—C28—C27	0.0 (5)
C1—N1—C14—C15	160.4 (2)	C26—C27—C28—C29	−0.6 (5)

### Hydrogen-bond geometry (Å, °)

**Table:**

*D*—H···*A*	*D*—H	H···*A*	*D*···*A*	*D*—H···*A*
N1—H1C···O4^i^	0.91	2.07	2.970 (3)	171
N4—H4D···O6^ii^	0.91	2.13	3.001 (3)	161
O3—H3B···O1^iii^	0.82	1.87	2.691 (3)	174
N3—H3C···O8	0.91	2.22	3.108 (3)	166
N2—H2C···O6^ii^	0.91	2.25	3.123 (3)	160

Symmetry codes: (i) −*x*+1, *y*+1/2, −*z*+3/2; (ii) *x*+1, *y*, *z*; (iii) −*x*+1, *y*−1/2, −*z*+3/2.

## References

[bb1] Basiuk, E. V., Basiuk, V. A., Hernández-Ortega, S., Martínez-García, M. & Saniger-Blesa, J.-M. (2001). *Acta Cryst.* C**57**, 553–555. 10.1107/s010827010100333x 11353247

[bb2] Bruker (1997). *SMART* Bruker AXS Inc., Madison, Wisconsin, USA.

[bb3] Bruker (2003). *SAINT* Bruker AXS Inc., Madison, Wisconsin, USA.

[bb4] Curtis, N. F. (1965). *J. Chem. Soc. A*, pp. 924–931.

[bb5] Jiang, L., Feng, X. L. & Lu, T. B. (2005). *Cryst. Growth Des.* **5**, 1469–1475.

[bb6] Ou, G.-C., Zhang, M. & Yuan, X.-Y. (2008*a*). *Acta Cryst.* E**64**, m1010. 10.1107/S1600536808020564 PMC2961933 21203004

[bb8] Sheldrick, G. M. (1996). *SADABS* University of Göttingen, Germany.

[bb9] Sheldrick, G. M. (2008). *Acta Cryst.* A**64**, 112–122. 10.1107/S0108767307043930 18156677

[bb10] Tait, A. M. & Busch, D. H. (1976). *Inorg. Synth.* **18**, 4–7.

